# From identical S- and P-wave $p_\mathrm{T}$ spectra to maximally distinct polarizations: probing NRQCD with $\chi$ states

**DOI:** 10.1140/epjc/s10052-018-5755-7

**Published:** 2018-03-29

**Authors:** Pietro Faccioli, Carlos Lourenço, Mariana Araújo, João Seixas, Ilse Krätschmer, Valentin Knünz

**Affiliations:** 1 LIP and IST, Lisbon, Portugal; 2 CERN, Geneva, Switzerland; 3 HEPHY, Vienna, Austria

## Abstract

A global analysis of ATLAS and CMS measurements reveals that, at mid-rapidity, the directly-produced $\chi _{c1}$, $\chi _{c2}$ and J/$\psi$ mesons have differential cross sections of seemingly identical shapes, when presented as a function of the mass-rescaled transverse momentum, $p_\mathrm{T}/M$. This identity of kinematic behaviours among S- and P-wave quarkonia is certainly not a natural expectation of non-relativistic QCD (NRQCD), where each quarkonium state is supposed to reflect a specific family of elementary production processes, of significantly different $p_\mathrm{T}$-differential cross sections. Remarkably, accurate kinematic cancellations among the various NRQCD terms (colour singlets and octets) of its factorization expansion can lead to a surprisingly good description of the data. This peculiar tuning of the NRQCD mixtures leads to a clear prediction regarding the $\chi _{c1}$ and $\chi _{c2}$ polarizations, the only observables not yet measured: they should be almost maximally different from one another, and from the J/$\psi$ polarization, a striking exception in the global panorama of quarkonium production. Measurements of the difference between the $\chi _{c1}$, $\chi _{c2}$ and J/$\psi$ polarizations, complementing the observed identity of momentum dependences, represent a decisive probe of NRQCD.

## Introduction

The mechanisms behind hadron production continue to challenge our understanding: analytical perturbative QCD calculations are insufficient to tackle all the aspects of the strong interactions driving the binding of quarks into observable particles. Studies of quarkonium production can provide crucial progress towards solving this problem [1]. According to non-relativistic QCD (NRQCD) [2], one of the theory approaches in this area of QCD phenomenology, S- and P-wave quarkonia are produced from the binding of quark-antiquark pairs created with a variety of quantum numbers, in color singlet or octet configurations. These terms are characterized by significantly different kinematic dependences and polarizations, determined by the short-distance cross sections (SDCs), presently calculated at next-to-leading order (NLO) [3–6]. They contribute with probabilities proportional to long distance matrix elements (LDMEs), extracted from fits to experimental data. While conceptually appealing and successful in several respects, it has been confusing to see that different groups performing global fits to experimental data extract significantly different matrix elements, despite using identical theory calculations, as a result of using different data fitting strategies [3–6]. These puzzles and a potential solution were discussed in Ref. [7], mostly devoted to the quarkonia least affected by feed-down decays from heavier quarkonia, the $\psi \mathrm{(2S)}$ and $\Upsilon$(3S) states. A detailed data-driven analysis of the cross sections and polarizations of five S-wave and two P-wave states, complemented by an original comparison with theory calculations, was presented in Ref. [8]. That analysis is extended in this paper to address two main questions: how different and experimentally recognizable are the $\chi _c$ production mechanisms with respect to those of the $\mathrm{J}/\psi$ and $\psi \mathrm{(2S)}$ mesons; and to what extent new or improved $\chi _c$ measurements will be important in the understanding of quarkonium production.

## Data-driven considerations

**Fig. 1:**
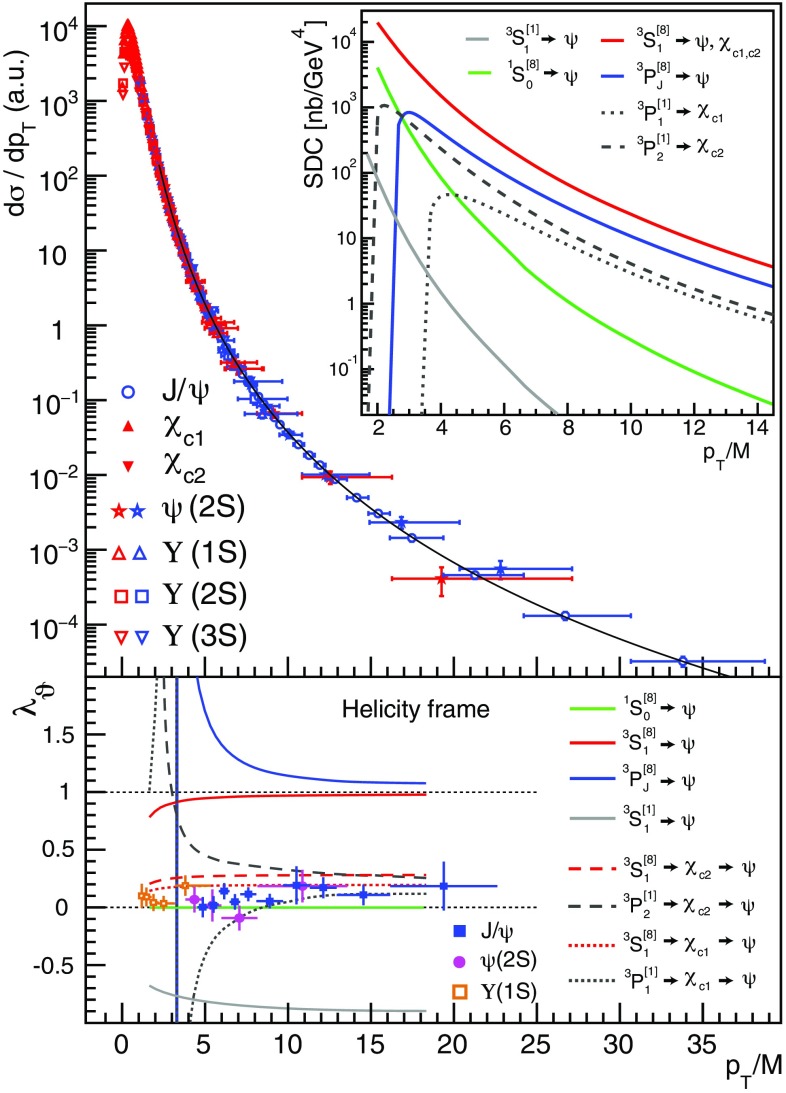
Top: mid-rapidity prompt quarkonium cross sections measured in pp collisions at $\sqrt{s} = 7$ TeV by ATLAS (red markers) [9–11] and CMS (blue markers) [12, 13]. The normalizations were adjusted to the $\mathrm{J}/\psi$ points to directly illustrate the universality of the $p_\mathrm{T}/M$ dependence. The curve represents a fit to all points of $p_\mathrm{T}/M > 2$ [8], with a normalized $\chi ^2$ of $215/193 = 1.11$. The inset shows the NLO SDCs [5, 14, 15]. The ${^3\mathrm{P}_J^{[8]}}$ and ${^3\mathrm{P}_{1,2}^{[1]}}$ SDCs are multiplied by $m_c^2$, the mass of the charm quark squared; they are negative and plotted with flipped signs. Bottom: polar anisotropy parameter $\lambda _\vartheta$, in the helicity frame, measured by CMS in pp collisions at $\sqrt{s} = 7$ TeV, for prompt $\mathrm{J}/\psi$, $\psi \mathrm{(2S)}$ and $\Upsilon \mathrm{(1S)}$ dimuon decays [16, 17]. For improved visibility, values corresponding to two or three rapidity bins were averaged. The curves represent the calculated $\lambda _\vartheta = (\mathcal {S}_\mathrm{T}-\mathcal {S}_\mathrm{L}) / (\mathcal {S}_\mathrm{T}+\mathcal {S}_\mathrm{L})$ values, where $\mathcal {S}_\mathrm{T}$ ($\mathcal {S}_\mathrm{L}$) is the transverse (longitudinal) short distance cross section, in the helicity frame (HX)

As shown in the top panel of Fig. 1, the ${^3\mathrm{S}_1}$ and ${^3\mathrm{P}_J}$ charmonium and bottomonium cross sections measured at the LHC at mid-rapidity show a remarkably uniform pattern as a function of $p_\mathrm{T}/M$, the ratio between the quarkonium transverse momentum and its mass (Fig. 1 of Ref. [8] shows the seven independent distributions). Moreover, the corresponding measurements of the quarkonium decay distributions indicate similar polarizations for all S-wave states, independently of their different P-wave feed-down contributions, as expressed by their polar anisotropy parameters, in the helicity frame, shown in the bottom panel.

This seemingly “universal” picture of quarkonium production is an unexpected result, when compared to the wide variety of kinematic shapes of the differential cross sections (NLO SDCs) contributing to the observable patterns within the NRQCD framework, as shown in the inset of Fig. 1. The most surprising aspect is that the $\chi _{c1}$ and $\chi _{c2}$ P-wave states have, at least at mid-rapidity, $p_\mathrm{T}/M$ distribution shapes indistinguishable from those of the S-wave states. According to the SDCs calculated at NLO, on the other hand, the singlet and octet P-wave terms of the NRQCD expansion, which contribute differently to $\mathrm{J}/\psi$ ($\psi \mathrm{(2S)}$), $\chi _{c1}$ and $\chi _{c2}$ production, have rather peculiar and differentiated kinematic behaviours, with cross section terms becoming negative above characteristic $p_\mathrm{T}/M$ thresholds and having unphysical polarization parameters ($|\lambda _\vartheta |>1$). In advance of any detailed numerical analysis, the qualitative comparison between data and theory illustrated in Fig. 1 indicates that the theory requires precise and seemingly unnatural cancellations between terms of the expansion, in order to reproduce observable cross sections and polarizations that are not only physical but also identical for states of different quantum numbers.

It should be noted that comparing the shapes of seven different quarkonium states, including five S-wave states affected by very different fractions of P-wave feed-down contributions, provides a stronger (more precise) statement regarding the overall equality between S- and P-wave quarkonium production than one might initially expect, given the uncertainties of the $\chi _{c1}$ and $\chi _{c2}$ measurements on their own. We will quantify this observation when presenting the results of a global fit to all charmonium data.

**Fig. 2:**
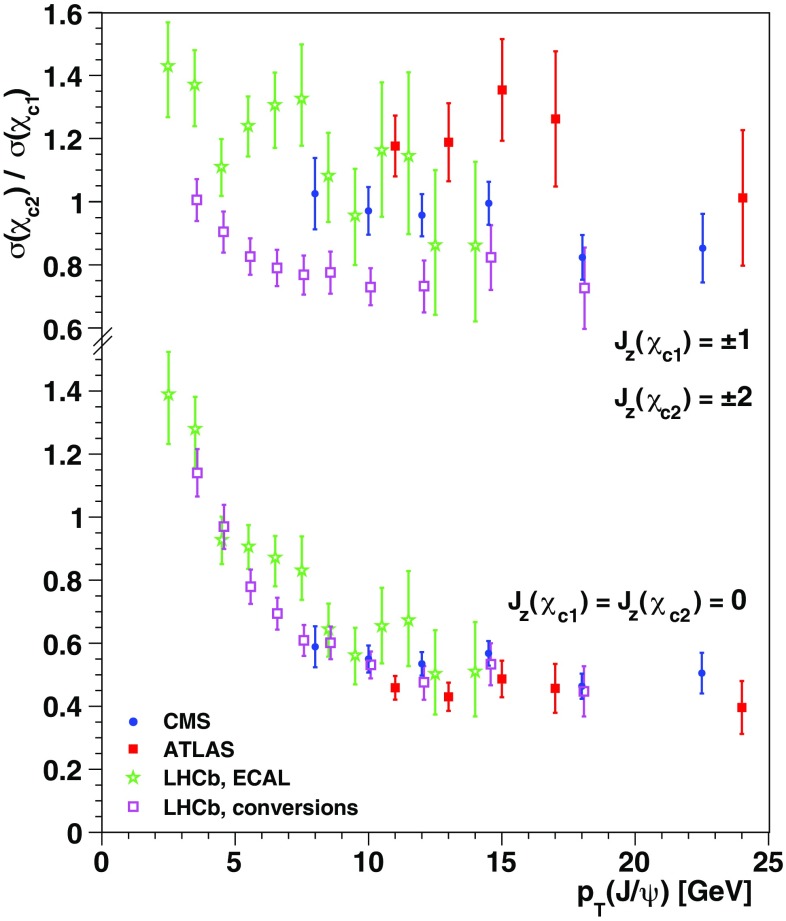
The $\chi _{c2}/\chi _{c1}$ ratio measured in pp collisions at 7 TeV by ATLAS [11], CMS [20] and LHCb [21, 22], with acceptance corrections calculated with two extreme polarization hypotheses: spin alignments $J_z(\chi _{c1}) = \pm 1$, $J_z(\chi _{c2}) = \pm 2$ (top) and $J_z(\chi _{c1}) = J_z(\chi _{c2}) = 0$ (bottom). The unpolarized hypothesis leads to intermediate values

The $\chi _{c1}$ and $\chi _{c2}$ polarizations are the main missing element in the current experimental landscape, but two data-driven observations provide indirect indications. First, the $\psi \mathrm{(2S)}$, $\mathrm{J}/\psi$ and $\Upsilon \mathrm{(1S)}$ polarizations are very similar (Fig. 1bottom), despite the diversity of $\chi$ feed-down fractions (0, $\sim$ 25% [11, 18] and $\sim$ 40% [19], respectively). Assuming that the *directly-produced* S-wave mesons have very similar production mechanisms, as indicated by the seemingly identical shapes of the $p_\mathrm{T}/M$-differential cross sections (Fig. 1top), the $\chi _{c1}$ plus $\chi _{c2}$ summed feed-down contributions cannot have a large impact in the observed $\mathrm{J}/\psi$ polarization. The second observation derives from comparing $\chi _{c2}/\chi _{c1}$ cross-section ratios measured in different experimental acceptances, profiting from their strong sensitivity to the polarization hypothesis used in the acceptance corrections. Interestingly, as seen in Fig. 2, the $J_z = 0$ alignment hypothesis gives the best mutual agreement between the $\chi _{c2}/\chi _{c1}$ ratios reported by ATLAS and CMS, as well as between the LHCb values obtained using photons detected in the calorimeter or with conversions to $e^+e^-$ pairs. When both are polarized in the $J_z = 0$ limit, the $\chi _{c1}$ and $\chi _{c2}$ decays produce strongly polarized $\mathrm{J}/\psi$ mesons, with, respectively, $\lambda _\vartheta = +1$ and $\lambda _\vartheta = - 3/5$, leading to a weighted $\lambda _\vartheta \sim 0.3$ when the feed-down fractions and the cross-section ratio itself are taken into account. These observations suggest that the $\chi _{c1}$ and $\chi _{c2}$ polarizations might be a striking exception in the global panorama of high-energy quarkonium production, at least at mid rapidity.

## Analysis method

To quantify our previous data-driven considerations and compare the results with theory, we perform a simultaneous fit of the mid-rapidity differential cross sections and polarizations, including a detailed account of how the mother’s momentum and polarization are transferred to the daughter in the relevant feed-down decays: $\psi \mathrm{(2S)} \rightarrow \chi _{c1,2} \; \gamma$; $\psi \mathrm{(2S)} \rightarrow \mathrm{J}/\psi \; X$; $\chi _{c1,2} \rightarrow \mathrm{J}/\psi \; \gamma$. The analysis is restricted to the charmonium family, given the lack of experimental information on bottomonium feed-down fractions. The rule for the momentum propagation from mother to daughter is, approximately, $p_\mathrm{T}/m = P_\mathrm{T}/M$, where *M* (*m*) and $P_\mathrm{T}$ ($p_\mathrm{T}$) are, respectively, the mass and laboratory transverse momentum of the mother (daughter) particle [8]. The polarization transfer rules were calculated in the electric dipole approximation and precisely account for the observable dilepton distribution with no need of higher-order terms [23]. The fit is exclusively based on empirical parametrizations. Perturbative calculations of the production kinematics are not used as ingredients anywhere in our analysis, the outcome of the fit being exclusively determined by the measurements and, therefore, only affected by statistical and systematic experimental uncertainties.

Inspired by the pattern of slightly transverse polarizations seen in Fig. 1, we parametrize the directly-produced $\mathrm{J}/\psi$ and $\psi \mathrm{(2S)}$ cross section shapes as a superposition of unpolarized ($\lambda _\vartheta =0$) and transversely polarized ($\lambda _\vartheta =+1$) processes, $\lambda _\vartheta$ being the polar anisotropy parameter of the dilepton decay in the helicity frame [24]: $\sigma _\mathrm{dir} \propto [ (1-f_\mathrm{p}) \, g_\mathrm{u} + f_\mathrm{p} \, g_\mathrm{p} ]$, where $f_\mathrm{p}$, identical for the two charmonia, is the fractional contribution of the polarized process considered at an arbitrary reference point $(p_\mathrm{T}/M)^{*}$. The shape functions $g_\mathrm{u}(p_\mathrm{T}/M)$ and $g_\mathrm{p}(p_\mathrm{T}/M)$ describe the $p_\mathrm{T}/M$ dependences of, respectively, the unpolarized and polarized yields. Both are normalized to unity at the chosen $(p_\mathrm{T}/M)^{*}$: $g(p_\mathrm{T}/M) = h(p_\mathrm{T}/M) / h((p_\mathrm{T}/M)^{*})$, with

1 $$\begin{aligned} h(p_\mathrm{T}/M) = \frac{p_\mathrm{T}}{M} \cdot \left( 1+\frac{1}{\beta -2} \cdot \frac{(p_\mathrm{T}/M)^2}{\gamma }\right) ^{-\beta }. \end{aligned}$$

The parameter $\gamma$ (having the meaning of the average $p_\mathrm{T}/M$ squared) defines the function in the low-$p_\mathrm{T}$ turn-on region and is only mildly sensitive to the data we are considering here; hence, in the fit we consider $\gamma$ as a common free parameter. The $\beta$ power-law exponent, instead, characterizes the high-$p_\mathrm{T}$ shape: $h \propto (p_\mathrm{T}/M)^{1-2\beta }$ for $p_\mathrm{T}/M \gg \sqrt{\gamma (\beta -2)}$. Therefore, we distinguish the unpolarized and polarized cross sections with two different powers, $\beta _\mathrm{u}$ and $\beta _\mathrm{p}$, respectively, identical for the $\mathrm{J}/\psi$ and $\psi \mathrm{(2S)}$. The relative contributions and shapes of the $g_\mathrm{u}$ and $g_\mathrm{p}$ functions are constrained by the polarization data. In fact, the polarized yield fraction, equal to $f_\mathrm{p}$ at $(p_\mathrm{T}/M)^{*}$, can be expressed as a function of $p_\mathrm{T}/M$ as $3 \lambda _\vartheta (p_\mathrm{T}/M) / [4 - \lambda _\vartheta (p_\mathrm{T}/M) ]$.

For the $\chi _{c1}$ and $\chi _{c2}$ direct cross sections we use the same general $p_\mathrm{T}/M$ shape parametrization, but without discriminating between polarized and unpolarized contributions, which, in the absence of $\chi$ polarization data, would not be individually constrained by the fit. In short, we consider four contributions to direct quarkonium production, the unpolarized and polarized $\psi$ terms plus the $\chi _{c1}$ and $\chi _{c2}$ cross sections, altogether characterized by one $\gamma$ and four $\beta$ parameters, $\beta _\mathrm{u}$, $\beta _\mathrm{p}$, $\beta (\chi _1)$ and $\beta (\chi _2)$. Their theoretical counterparts are, respectively, ${^1\mathrm{S}_0^{[8]}}$, ${^3\mathrm{S}_1^{[8]}} +{^3\mathrm{P}_J^{[8]}}$, ${^3\mathrm{S}_1^{[8]}} +{^3\mathrm{P}_{1}^{[1]}}$ and ${^3\mathrm{S}_1^{[8]}} +{^3\mathrm{P}_{2}^{[1]}}$ (where each term indicates the SDC function times the LDME constant), the four leading cross section components foreseen by NRQCD hierarchies for ${^3\mathrm{S}_1}$ and ${^3\mathrm{P}_J}$ quarkonium production. However, this parallelism is only a guidance in the parametrization of the fit, not a theoretical input. As discussed in more detail in Ref. [8], our approach is very different with respect to fits using the calculated SDC shapes, where the fit results are mostly determined by the $p_\mathrm{T}$-differential cross sections; the less precise polarization data are not included in the fits or have a negligible effect. In our fit, the polarization data, versus $p_\mathrm{T}/M$, have the exclusive role of constraining both the relative normalizations and the differences in momentum dependence of the polarized and unpolarized contributions. The precision of these data-driven results will evolve as new measurements become available, remaining insensitive to specific theoretical calculations and uncertainties.

Without $\chi$ polarization measurements, the current $\chi _{c1}$ and $\chi _{c2}$ cross-section data cannot discriminate between the $g_u$ and $g_p$ contributions to $\mathrm{J}/\psi$ production and, therefore, relate the parametrized direct-$\mathrm{J}/\psi$ polarization to the measured prompt one. However, the two data-driven observations mentioned above allow us to implement such a relation by adopting an approximate constraint on the total $\chi _{c}$ polarization contribution to $\mathrm{J}/\psi$ production. Given that, on average, $\lambda _\vartheta ^{\mathrm{J}/\psi } \gtrsim \lambda _\vartheta ^{\psi \mathrm{(2S)}}$, we can infer that $\lambda _\vartheta ^{\mathrm{J}/\psi \leftarrow \chi _c}$ should be positive, under the assumption that the direct $\mathrm{J}/\psi$ and $\psi \mathrm{(2S)}$ polarizations are equal. On the other hand, the extreme hypothesis, discussed above, according to which both $\chi _{c1}$ and $\chi _{c2}$ are polarized in the $J_z = 0$ limit leads to $\lambda _\vartheta ^{\mathrm{J}/\psi \leftarrow \chi _c} \simeq 0.3$ (which, when weighted by the 25% feed-down fraction of $\mathrm{J}/\psi$ from $\chi _c$, is comparable to the average difference $\lambda _\vartheta ^{\mathrm{J}/\psi } - \lambda _\vartheta ^{\psi \mathrm{(2S)}} \simeq 0.05$). We can thus be confident that $\lambda _\vartheta ^{\mathrm{J}/\psi \leftarrow \chi _c}$ is positive and not larger than 0.3. The results of the fit, and ensuing considerations, are insensitive to variations of $\lambda _\vartheta ^{\mathrm{J}/\psi \leftarrow \chi _c}$ (assumed to be $p_\mathrm{T}/M$-independent) within this range.

The ATLAS and CMS integrated-luminosity uncertainties are (independently) varied as nuisance parameters, following Gaussian functions centred at unity and of widths equal to the relative uncertainties of the published luminosities. These two nuisance parameters multiply all the data points (cross sections) of the respective experiment, thereby correlating the several datasets within each experiment. Moreover, the experiments measured products of cross sections times branching ratios, so that the uncertainties of the branching ratios have also been treated as nuisance parameters (with central values and uncertainties taken from Ref. [25]), multiplying all relevant data points and representing correlations between ATLAS and CMS.

Another source of correlation between all the points being fitted is the dependence of the detection acceptances on the polarization. For each set of parameter values considered in the fit scan, the expected values of the polarizations and cross sections are calculated, for all states, as functions of $p_\mathrm{T}$, using the shape-parametrization functions described above. The expected $\lambda _\vartheta$ values can be immediately compared to the measured ones, for the determination of the corresponding $\chi ^2$ terms, while for the calculation of the cross-section $\chi ^2$ terms we first scale the measured cross sections by acceptance-correction factors calculated for the $\lambda _\vartheta$ value under consideration. These correction factors are computed, for each data point, using the tables published by the experiments (for exactly this purpose) for the cross sections of particles produced with fully transverse or fully longitudinal polarization.

The fit has 100 experimental constraints and 20 parameters: 5 shape parameters, 4 normalizations and the fraction $f_p$, plus 2 luminosity and 8 branching-ratio nuisance parameters.

## Analysis results

**Fig. 3:**
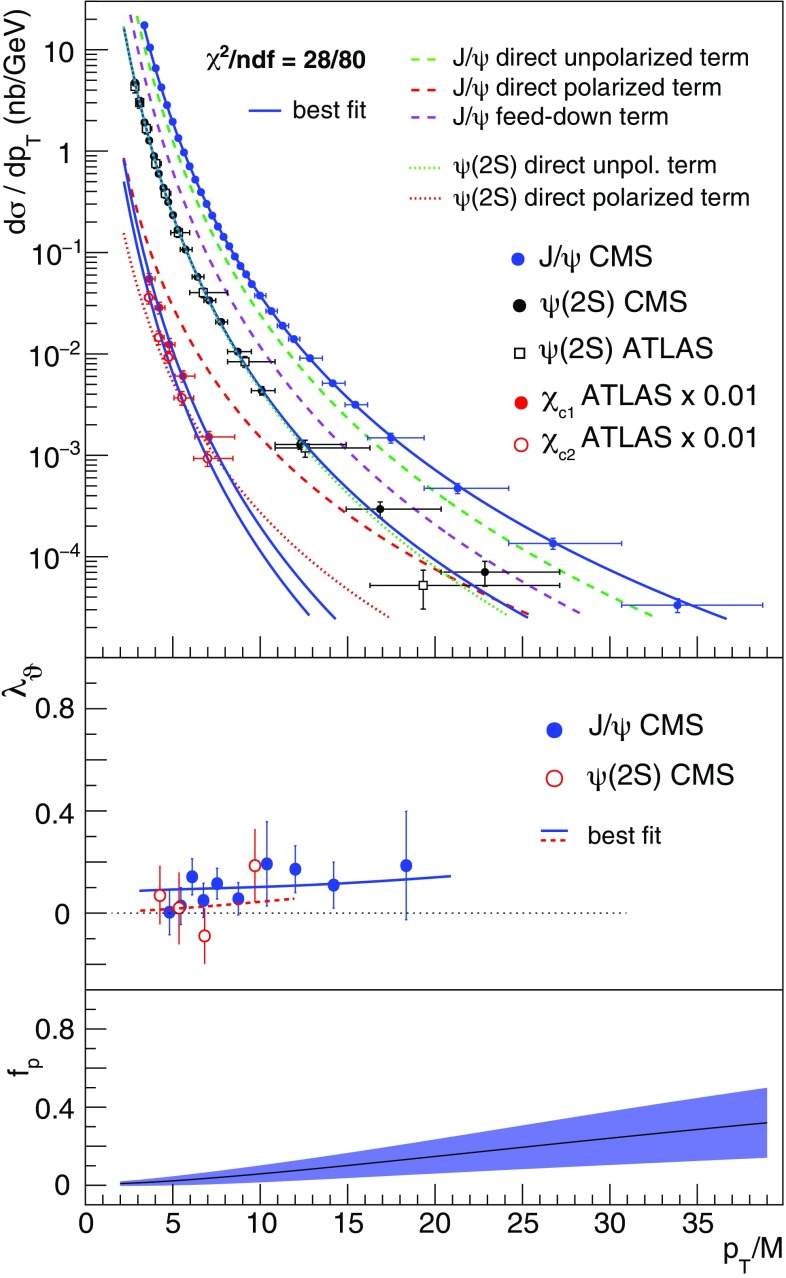
Comparison between the data and the fitted curves, for the $\mathrm{J}/\psi$, $\psi \mathrm{(2S)}$, $\chi _{c1}$ and $\chi _{c2}$ cross sections (top) and for the $\mathrm{J}/\psi$ and $\psi \mathrm{(2S)}$ polarizations (middle). The bottom panel shows the resulting $\psi$ polarized fraction

As shown in Fig. 3, the charmonium cross sections and polarizations are described by the fit just presented, with a $\chi ^2$ per degree of freedom of 28/80.

**Fig. 4:**
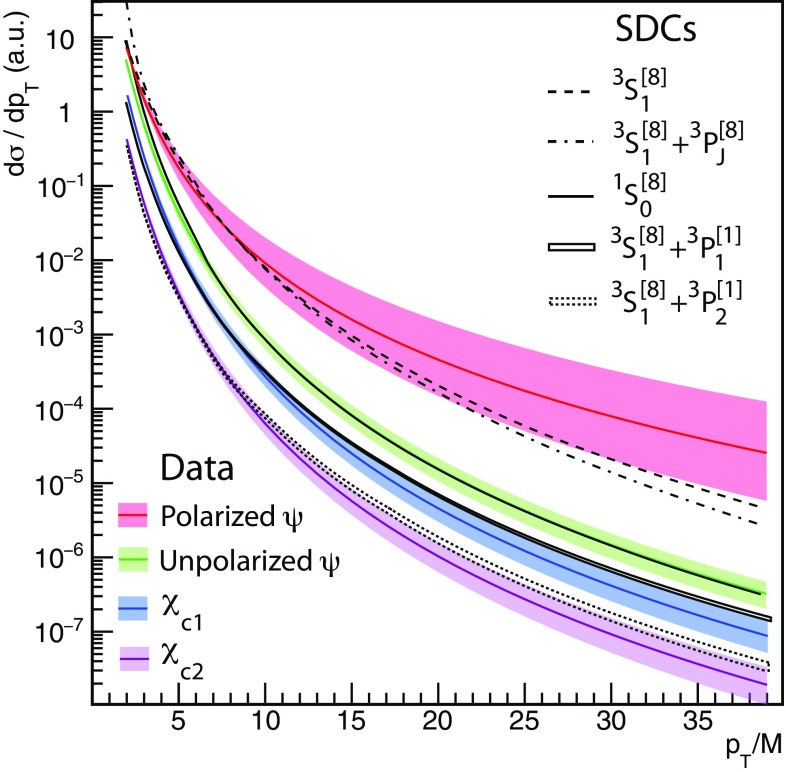
Direct production cross sections resulting from the fit of the data, with 68.3% confidence level uncertainty bands reflecting correlated variations in the fit parameters. The normalizations of the four bands are chosen for visibility reasons. Suitable SDC combinations are also shown, normalized to the respective bands at $p_\mathrm{T}/M = 8$. The widths of the $\chi _{c1}$ and $\chi _{c2}$ SDC bands reflect the 1.3% uncertainty of $K_{\chi }$ (see text)

Figure 4 shows the fitted cross section terms as bands of widths reflecting the experimental uncertainties.

A very interesting and non-trivial indication of this purely data-driven fit is that the $\chi _{c1}$ and $\chi _{c2}$ $p_\mathrm{T}/M$ distributions are very similar to the unpolarized term dominating $\psi$ production, as quantified by the compatibility of the $\beta$ parameters: $\beta _\mathrm{u} = 3.42 \pm 0.05$, $\beta (\chi _1) = 3.46 \pm 0.08$ and $\beta (\chi _2) = 3.49 \pm 0.10$. This very clear experimental observation is predominantly the result of the perfect compatibility of the (high precision) $\mathrm{J}/\psi$ and $\psi \mathrm{(2S)}$ $p_\mathrm{T}/M$ shapes, even in $p_\mathrm{T}/M$ ranges beyond those covered by the existing $\chi$ data, reflecting the fact that the prompt $\psi \mathrm{(2S)}$ mesons are fully directly produced while $\simeq 25\%$ [11] of the $\mathrm{J}/\psi$ yield comes from $\chi _{c}$ decays. In fact, the $\chi _{c}$ cross sections are measured at relatively low $p_\mathrm{T}$ and with comparatively poor precision. To verify this conclusion, we repeated the fit keeping only one experimental point for each of the two $\chi _{c}$ cross sections (chosen in the middle of the measured range), so that these measurements constrain the feed-down fractions at that point but not the $p_\mathrm{T}/M$ shapes. As expected, the fit results for the $\chi _{c}$ cross section shapes do not change significantly, with shape parameters remaining the same within the one-sigma range.

The experimental bands for the four observable cross sections are compared with the corresponding NRQCD terms, the dashed/dotted lines corresponding to (combinations of) SDCs calculated at NLO [5, 14, 15]. We emphasize that the two terms of comparison are completely independent, the first being the result of a model-independent fit of experimental data and the second a pure theoretical calculation. The unpolarized and polarized $\psi$ bands are compared with, respectively, the ${^1\mathrm{S}_0^{[8]}}$ and ${^3\mathrm{S}_1^{[8]}}$ SDC shapes, calculated at NLO and also including fragmentation corrections representing a partial account of next-to-next-to-leading order processes [26, 27]. Adding a ${^3\mathrm{P}_J^{[8]}}$ contribution to the ${^3\mathrm{S}_1^{[8]}}$ term leads to steeper shapes, increasing the departure from the polarized $\psi$ experimental band, so that the present measurements indicate that the ${^3\mathrm{P}_J^{[8]}}$ SDC has a negligible effect. This observation is qualitatively illustrated by the dot-dashed line, corresponding to $(1/{m_c^2}) \; \langle \mathcal{O}^{\mathrm{J}/\psi } ({^3\mathrm{P}_0^{[8]}}) \rangle = 0.1 \cdot \langle \mathcal{O}^{\mathrm{J}/\psi } ({^3\mathrm{S}_1^{[8]}}) \rangle$. The ${^1\mathrm{S}_0^{[8]}}$ SDC shape is in remarkable agreement with the experimental “unpolarized” band. Moreover, adding the negative ${^3\mathrm{P}_{1,2}^{[1]}}$ SDCs to the ${^3\mathrm{S}_1^{[8]}}$ term results in shapes approximating the ${^1\mathrm{S}_0^{[8]}}$ term, reproducing relatively well the observed similarity between the unpolarized-$\psi$ and $\chi _{c1,2}$ patterns.

## Predicted $\chi _c$ cross sections and polarizations

Before discussing in more detail the data-theory comparison, we need to explain how we derived the predictions for the $\chi _c$ differential cross sections and polarizations.

In NRQCD the $\chi _{c1,2}$ polarizations and cross sections are functions of one common parameter, equal for all $\chi _c$ states,

2 $$\begin{aligned} K_{\chi } = ({1}/{m_c^2}) \, \left\langle \mathcal{O}^{\chi _{c0}} \left( {^3\mathrm{P}_0^{[1]}}\right) \right\rangle \Big / \left\langle \mathcal{O}^{\chi _{c0}} \left( {^3\mathrm{S}_1^{[8]}}\right) \right\rangle , \end{aligned}$$

with $\langle \mathcal{O} \rangle$ denoting the LDME. The $\chi _c$ production cross sections $\sigma _J$ and the spin-density matrix elements $\sigma _J^{ij}$ have the general form

3 $$\begin{aligned} \sigma _J^{(ij)} \propto (2J+1) \left[ \mathcal {S}^{(ij)}\left( {^3\mathrm{S}_1^{[8]}}\right) + K_{\chi } \, m_c^2 \,\mathcal {S}^{(ij)}\left( {^3\mathrm{P}_J^{[1]}}\right) \right] ,\nonumber \\ \end{aligned}$$

where $\mathcal {S}^{(ij)}$ denotes the SDC or its spin projection. The $\lambda _\vartheta$ are calculated as $\lambda _\vartheta ^{\chi _1} = ( \sigma _1^{00} - \sigma _1^{11} ) / ( \sigma _1^{00} + 3 \sigma _1^{11} )$ and $\lambda _\vartheta ^{\chi _2} = ( -3 \sigma _2^{00} - 3 \sigma _2^{11} + 6 \sigma _2^{22} ) / ( 5 \sigma _2^{00} + 9 \sigma _2^{11} + 6 \sigma _2^{22} )$, where the $\sigma _J^{ij}$ depend on $K_{\chi }$ through Eq. 3. The $\chi _{c1,2}$ $\lambda _\vartheta$ parameters refer to the corresponding $\mathrm{J}/\psi$ dilepton decay distributions, which are the ones directly measured and fully reflect the $\chi$ polarization state, while being insensitive to the uncertain contributions of higher-order photon multipoles [23].

We determine $K_{\chi }$ from the $\chi _{c2}/\chi _{c1}$ ratios measured by ATLAS [11] and CMS [20], taking into account that the published values strongly depend on the $\chi _{c1}$ and $\chi _{c2}$ polarizations assumed for the corrections of the detector’s acceptance. We continuously vary the $K_{\chi }$ parameter and, for each value, we calculate the $\chi _{c1}$ and $\chi _{c2}$ polarizations using NLO SDCs and correct the published ratio by the corresponding acceptance ratio, using the correction tables provided in the experimental publications. The fit $\chi ^2$ is then calculated comparing the corrected measurement (with statistical and systematic uncertainties, but no “polarization uncertainties”) with the prediction for that $K_{\chi }$ value. The resulting fit $\chi ^2$ profile provides the $K_{\chi }$ central value and its uncertainty.

**Fig. 5:**
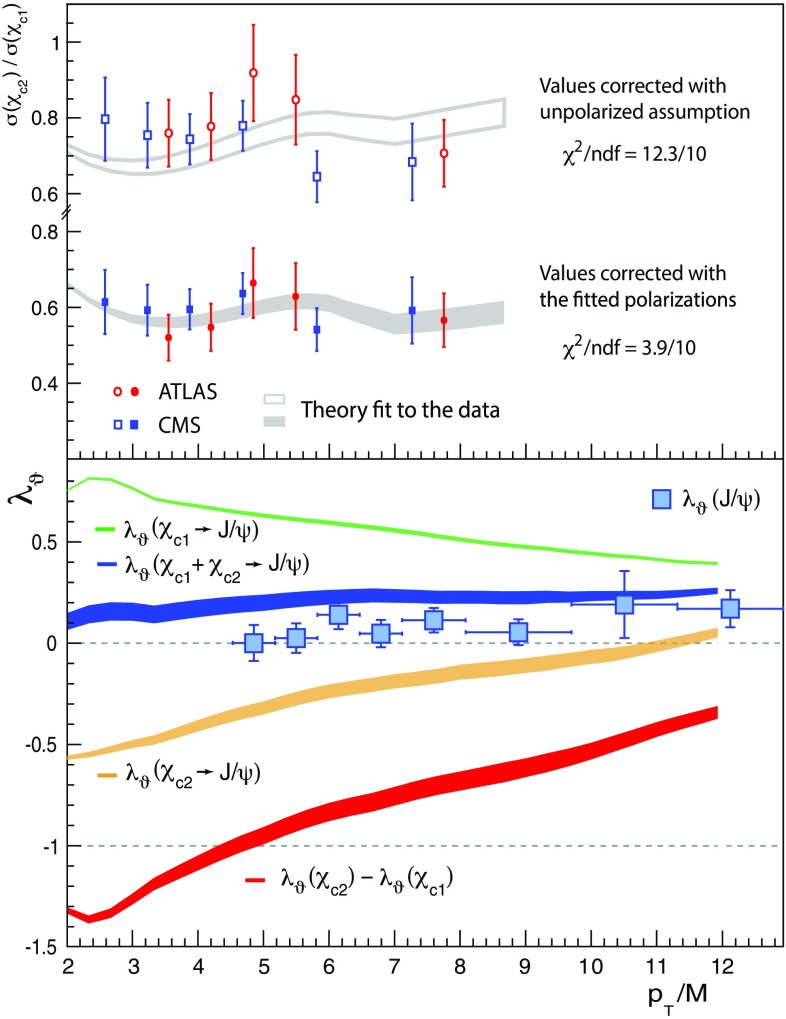
Top: $\chi _{c2}/\chi _{c1}$ ratio measured by ATLAS [11] and CMS [20], with acceptance corrections assuming unpolarized production (open markers) and “best-fit polarizations” (filled markers). The grey bands reflect the corresponding theory fits. Bottom: the best-fit $\chi _{c1,2}$ polarizations. In both panels, the widths of the bands reflect the uncertainty of $K_{\chi }$, the only free parameter of the fit

Figure 5top shows, as open symbols, the $\chi _{c2}/\chi _{c1}$ ratios reported by the experiments with acceptance corrections computed assuming unpolarized $\chi _{c1,2}$ production. The corresponding theory fit, shown by the open grey band, does not provide a satisfactory representation of the measurements. The theoretical fit improves considerably when the unpolarized scenario is replaced by the NRQCD polarization conjecture, with a free $K_{\chi }$ parameter. The result of our fit is $K_{\chi } = 4.60 \pm 0.06$, much more precise than the value $3.7^{+1.0}_{-0.7}$, derived in Ref. [14] under the scenario of unpolarized ratios and including the entire spectrum of polarization hypotheses in the experimental uncertainty.

The corresponding polarization predictions are shown in Fig. 5bottom. Interestingly, as $p_\mathrm{T}/M$ decreases, $\lambda _\vartheta$ tends to the extreme physical values $+1$ ($\chi _{c1}$) and $-3/5$ ($\chi _{c2}$), in agreement with the alignment scenario suggested by the measured $\chi _{c2}/\chi _{c1}$ cross-section ratios (Fig. 2): these limit values correspond to two very different decay distribution shapes, but to the same pure $J_z = 0$ angular momentum configuration of the $\chi _c$. The $\mathrm{J}/\psi$ $\lambda _\vartheta$ from the weighted $\chi _{c1}$ and $\chi _{c2}$ feed-downs (blue band) is close to the values measured by CMS (squares) for the prompt sample, implying that the direct and feed-down terms have similar polarizations.

It is quite remarkable to observe that the difference $\Delta \lambda _\vartheta \equiv \lambda _\vartheta (\chi _{c2}) - \lambda _\vartheta (\chi _{c1})$ is predicted with a rather high precision and, furthermore, reaches extreme values (around $-1$). In particular, in the region where experimental measurements will be provided in the near future, $p_\mathrm{T} \approx 20$ GeV ($p_\mathrm{T}/M \approx 6$), the prediction is $\Delta \lambda _\vartheta = -0.80 \pm 0.05$, implying a strong deviation from the mild polarizations shown in Fig. 1bottom. Comparing the discriminating power of this result to the corresponding predictions of Ref. [14] (Fig. 4), $\lambda _\vartheta (\chi _{c1}) = 0.25^{+0.08}_{-0.05}$ and $\lambda _\vartheta (\chi _{c2}) = 0.10^{+0.15}_{-0.20}$, one can see the crucial importance of a proper treatment of the uncertainties and correlations affecting the experimental data. It is also relevant to note that, thanks to the cancellation of most experimental systematic uncertainties, the *difference* $\lambda _\vartheta (\chi _{c2}) - \lambda _\vartheta (\chi _{c1})$ can be measured with maximal significance and accuracy.

## Discussion

**Fig. 6:**
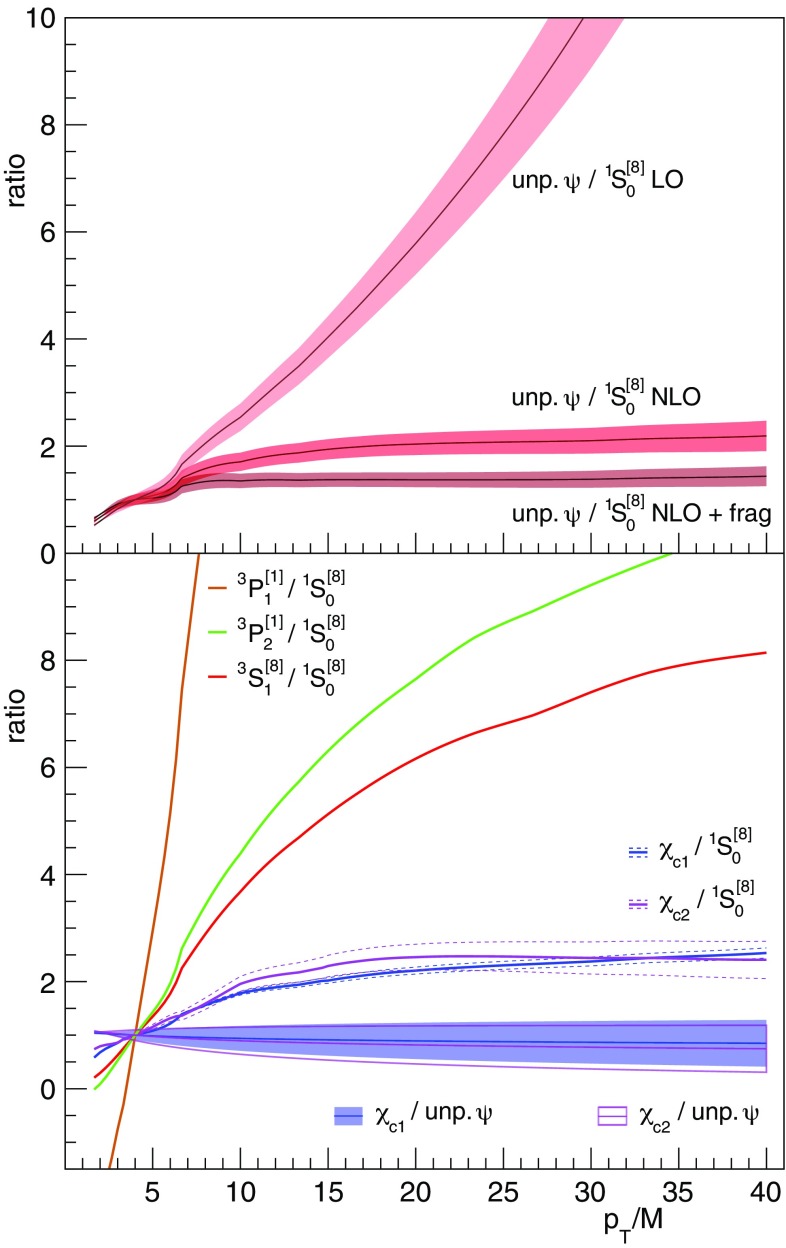
Ratios of direct-production charmonium cross section shapes for different combinations of the measured and/or calculated terms already presented in Fig. 4. For visibility reasons, all ratios are normalized to unity at $p_\mathrm{T}/M = 4$

We will now discuss in more detail the theory-data comparison. To discern shape differences more easily than in the logarithmic-scale plots of Fig. 4, we present in Fig. 6 some of the results in the form of ratios, in a linear scale.

In the top panel, we can see that the ratio between the experimental “unpolarized” band and the state-of-the-art ${^1\mathrm{S}_0^{[8]}}$ SDC curve (“NLO + frag.” band) only deviates from a perfectly flat function in the low-$p_\mathrm{T}/M$ region ($p_\mathrm{T}/M < 7$). This effect might represent a residual limitation of current finite-order perturbative calculations, as suggested by the observation that the ratio shows a more pronounced non-flatness when the SDC is calculated at NLO without fragmentation contributions (“NLO” band), and is not flat at all when we use the LO SDC as reference (“LO” band). The differences between these three ratios provide a pedagogical illustration of the improvements made in the successive evolutions of the calculations. The bottom panel shows that the ratios between the measured $\chi _{c1}$ (blue filled band) or $\chi _{c2}$ (pink open band) $p_\mathrm{T}/M$-differential cross sections and the corresponding unpolarized-$\psi$ cross section are practically identical to each other, and essentially flat, offering an effective representation of the strong experimental observation mentioned above. It is interesting to compare these two bands, exclusively determined by the measurements, with the two corresponding (and completely independent) theory ratios, here represented by the (blue and pink) solid lines, calculated as the ratios between suitable combinations of the ${^3\mathrm{S}_1^{[8]}}$ and ${^3\mathrm{P}_{1,2}^{[1]}}$ SDCs (analogous to the $\chi _{c1}$ or $\chi _{c2}$) and the ${^1\mathrm{S}_0^{[8]}}$ SDC (analogous to the unpolarized-$\psi$). The dashed curves surrounding the solid ones reflect the 1.3% uncertainty on $K_{\chi }$, already shown in Fig. 4. Also this ratio deviates from a flat function in the lower part of the $p_\mathrm{T}/M$ range, but this deviation is a relatively small effect, as can be judged by comparing it with the corresponding rate of increase of the *individual* components, ${^3\mathrm{S}_1^{[8]}}/ {^1\mathrm{S}_0^{[8]}}$ (red curve) and ${^3\mathrm{P}_{1,2}^{[1]}} / {^1\mathrm{S}_0^{[8]}}$ (brown and green curves). It is actually quite remarkable to see how effective is the mutual cancellation of the individual (steep) variations, in the combinations pertinent to the $\chi _{c1}$ and $\chi _{c2}$ states. Also taking into consideration that the P-wave SDCs seem to be affected by a slower convergence of the perturbative series than the S-wave SDCs [27], the present level of agreement between the shapes of the $\chi _{c}$-to-${^1\mathrm{S}_0^{[8]}}$ predicted ratios and the corresponding $\chi _{c}$-to-unpolarized-$\psi$ measured bands can be considered very promising. As a matter of fact, and despite the initial impression of unnecessary complexity expressed by Fig. 1, we see that NRQCD provides predictions that are, already today, very close to reproducing the uniformity of the observed $p_\mathrm{T}/M$ trends, as well as the small measured S-wave polarizations. This unexpected agreement is the result of a series of cancellations, which, given their fragile and unstable nature, must be tested with precise ingredients. Further improvements in the perturbative calculations, especially for the P-wave SDCs, are needed for more conclusive statements.

## Summary

The $\chi _{c1}$ and $\chi _{c2}$ states have, both, $p_\mathrm{T}/M$ distributions with shapes compatible to that of the $\mathrm{J}/\psi$ mesons. This conclusion results from the study of the full set of charmonium data and has a much higher significance than one would obtain if only considering the $\chi _{c}$ cross section measurements, given their limited precision and $p_\mathrm{T}$ coverage in comparison to the $\mathrm{J}/\psi$ and $\psi \mathrm{(2S)}$ measurements. This is a very specific and non-trivial experimental observation, seemingly in contradiction, at least a priori, with the expectations of NRQCD, given the significantly different shapes of the relevant SDCs. Remarkably, thanks to mutual cancellations of the steep SDC shapes differences, NLO NRQCD calculations approximately reproduce the similarity between the $\chi _{c1}$, $\chi _{c2}$ and $\psi$ cross sections shapes, giving a satisfactory description of charmonium production as measured at mid-rapidity by the ATLAS and CMS experiments. This happens in a very specific and non-trivial configuration, leading to a surprising prediction: the $\chi _{c1}$ and $\chi _{c2}$ polarizations are as different from each other as physically possible.

If confirmed experimentally, through an accurate measurement of the variable $\lambda _\vartheta (\chi _{c2}) - \lambda _\vartheta (\chi _{c1})$, the existence of strong $\chi _{c1}$ and $\chi _{c2}$ polarizations (an exception among all quarkonia observed by high-$p_\mathrm{T}$ experiments) would be a big step forward to confirm the existence of the diversified and polarized processes that are at the heart of NRQCD. If, instead, similar and weak $\chi _{c1}$ and $\chi _{c2}$ polarizations will be measured, it will be crucial to investigate if the predicted strong and opposite polarizations, experimentally falsified, are caused by approximations and inaccuracies of the presently available fixed-order perturbative calculations or from problems in the conceptual foundations of the theory. In that case, NRQCD would be facing a big challenge: even if future improvements of the P-wave SDC calculations would eventually make the $\chi _{c1}$ and $\chi _{c2}$ polarization predictions compatible with the measurements (e.g., building upon the recent progress on fragmentation corrections [27]) one would still think that the homogeneity of the observed kinematic patterns deserves a more natural theoretical explanation than a series of “coincidences” cancelling out the variegated complexity of NRQCD. In either case, accurate measurements of the $\chi _{c1}$ and $\chi _{c2}$ polarizations constitute a decisive test of NRQCD.

## References

[1] N. Brambilla et al., Eur. Phys. J. C **71**, 1534 (2011). 10.1140/epjc/s10052-010-1534-9. arXiv:1010.5827, and references therein

[2] G.T. Bodwin, E. Braaten, P. Lepage, Phys. Rev. D **51**, 1125 (1995). 10.1103/PhysRevD.51.1125. arXiv:hep-ph/9407339. [Erratum: Phys. Rev. D **55**, 5853 (1997)]

[3] M. Butenschön, B. Kniehl, Nucl. Phys. Proc. Suppl. **222**, 151 (2012). 10.1016/j.nuclphysbps.2012.03.016. arXiv:1201.3862

[4] Butenschön M, Kniehl Mod B (2013). Phys. Lett. A.

[5] Chao K-T (2012). Phys. Rev. Lett..

[6] Gong B, Wan L-P, Wang J-X, Zhang H-F (2013). Phys. Rev. Lett..

[7] Faccioli P (2014). Phys. Lett. B.

[8] Faccioli P (2017). Phys. Lett. B.

[9] ATLAS Coll, JHEP **09**, 079 (2014). 10.1007/JHEP09(2014)079. arXiv:1407.5532

[10] ATLAS Coll, Phys. Rev. D **87**, 052004 (2013). 10.1103/PhysRevD.87.052004. arXiv:1211.7255

[11] ATLAS Coll, JHEP **07**, 154 (2014). 10.1007/JHEP07(2014)154. arXiv:1404.7035

[12] CMS Coll, Phys. Rev. Lett. **114**, 191802 (2015). 10.1103/PhysRevLett.114.191802. arXiv:1502.04155

[13] CMS Coll, Phys. Lett. B **749**, 14 (2015). 10.1016/j.physletb.2015.07.037. arXiv:1501.07750

[14] Shao H-S, Ma Y-Q, Wang K, Chao K-T (2014). Phys. Rev. Lett..

[15] H.-S. Shao, Comput. Phys. Commun. **198**, 238 (2016). 10.1016/j.cpc.2015.09.011, arXiv:1507.03435

[16] CMS Coll, Phys. Lett. B **727**, 381 (2013). 10.1016/j.physletb.2013.10.055. arXiv:1307.6070

[17] CMS Coll, Phys. Rev. Lett. **110**, 081802 (2013). 10.1103/PhysRevLett.110.081802. arXiv:1209.2922

[18] P. Faccioli, C. Lourenço, J. Seixas, H. Wöhri, JHEP **10**, 004 (2008). 10.1088/1126-6708/2008/10/004. arXiv:0809.2153

[19] LHCb Coll, Eur. Phys. J. C **74**, 3092 (2014). 10.1140/epjc/s10052-014-3092-z. arXiv:1407.7734

[20] CMS Coll, Eur. Phys. J. C **72**, 2251 (2012). 10.1140/epjc/s10052-012-2251-3. arXiv:1210.0875

[21] LHCb Coll, Phys. Lett. B **714**, 215 (2012). 10.1016/j.physletb.2012.06.077. arXiv:1202.1080

[22] LHCb Coll, JHEP **10**, 115 (2013). 10.1007/JHEP10(2013)115. arXiv:1307.4285

[23] P. Faccioli, C. Lourenço, J. Seixas, H. Wöhri, Phys. Rev. D **83**, 096001 (2011). 10.1103/PhysRevD.83.096001. arXiv:1103.4882

[24] P. Faccioli, C. Lourenço, J. Seixas, H. Wöhri Eur. Phys. J. C **69**, 657 (2010). 10.1140/epjc/s10052-010-1420-5. arXiv:1006.2738

[25] Particle Data Group, Chin. Phys. C **40**, 100001 (2016). 10.1088/1674-1137/40/10/100001

[26] G.T. Bodwin, H.S. Chung, U.-R. Kim, J. Lee, Phys. Rev. Lett. **113**, 022001 (2014). 10.1103/PhysRevLett.113.022001. arXiv:1403.3612

[27] Bodwin GT (2016). Phys. Rev. D.

